# The non-linear and lagged short-term relationship between rainfall and leptospirosis and the intermediate role of floods in the Philippines

**DOI:** 10.1371/journal.pntd.0006331

**Published:** 2018-04-16

**Authors:** Naohiko Matsushita, Chris Fook Sheng Ng, Yoonhee Kim, Motoi Suzuki, Nobuo Saito, Koya Ariyoshi, Eumelia P. Salva, Efren M. Dimaano, Jose B. Villarama, Winston S. Go, Masahiro Hashizume

**Affiliations:** 1 Department of Paediatric Infectious Diseases, Institute of Tropical Medicine (NEKKEN), Nagasaki University. Nagasaki, Japan; 2 Graduate School of Biomedical Sciences, Nagasaki University, Nagasaki, Japan; 3 Department of Global Environmental Health, The School of Public Health, The University of Tokyo, Tokyo, Japan; 4 Department of Clinical Infectious Diseases, Institute of Tropical Medicine (NEKKEN), Nagasaki University, Nagasaki, Japan; 5 San Lazaro Hospital, Manila, Republic of the Philippines; University of California Berkeley, UNITED STATES

## Abstract

**Background:**

Leptospirosis is a worldwide bacterial zoonosis. Outbreaks of leptospirosis after heavy rainfall and flooding have been reported. However, few studies have formally quantified the effect of weather factors on leptospirosis incidence. We estimated the association between rainfall and leptospirosis cases in an urban setting in Manila, the Philippines, and examined the potential intermediate role of floods in this association.

**Methods/Principal findings:**

Relationships between rainfall and the weekly number of hospital admissions due to leptospirosis from 2001 to 2012 were analyzed using a distributed lag non-linear model in a quasi-Poisson regression framework, controlling for seasonally varying factors other than rainfall. The role of floods on the rainfall–leptospirosis relationship was examined using an indicator. We reported relative risks (RRs) by rainfall category based on the flood warning system in the country. The risk of post-rainfall leptospirosis peaked at a lag of 2 weeks (using 0 cm/week rainfall as the reference) with RRs of 1.30 (95% confidence interval: 0.99–1.70), 1.53 (1.12–2.09), 2.45 (1.80–3.33), 4.61 (3.30–6.43), and 13.77 (9.10–20.82) for light, moderate, heavy, intense and torrential rainfall (at 2, 5, 16, 32 and 63 cm/week), respectively. After adjusting for floods, RRs (at a lag of 2 weeks) decreased at higher rainfall levels suggesting that flood is on the causal pathway between rainfall and leptospirosis.

**Conclusions:**

Rainfall was strongly associated with increased hospital admission for leptospirosis at a lag of 2 weeks, and this association was explained in part by floods.

## Author summary

Leptospirosis is a worldwide bacterial zoonosis which is mainly transmitted through contact with water contaminated by rodents’ urine. It manifests with various symptoms, ranging from fever and muscle pain to a severe syndrome characterized by jaundice, renal failure and pulmonary hemorrhage. Outbreaks of leptospirosis after heavy rainfall and flooding have been reported, but few studies have evaluated the effect of weather factors on leptospirosis. We estimated the association between rainfall and leptospirosis cases in an urban setting in Manila, the Philippines, and examined the potential intermediate role of floods in this association. The risk of post-rainfall leptospirosis peaked at a lag of 2 weeks. After adjusting for floods, the effect of rainfall at lag 2 decreased at higher rainfall levels suggesting that flooding is on the causal pathway between heavy rainfall and leptospirosis. The results are useful for public health interventions to prepare hospitals and clinics for increased number of patients in case of an outbreak, which can help reduce the disease burden.

## Introduction

Leptospirosis is a worldwide bacterial zoonosis caused by pathogenic spirochetes of the genus *Leptospira*. The disease is most commonly transmitted through skin, especially abraded skin exposed to water contaminated with the urine of infected rodents or other wild or domesticated animals [1–3]. Globally, there are approximately one million cases of human leptospirosis with 58,900 related deaths occurring every year [4]. The disease is usually diagnosed clinically because diagnostic tests are often not available in many resource-limited settings [5, 6]. Clinical presentations of the disease can range from subclinical or mild fever, mimicking other types of febrile illness such as dengue, typhoid fever and pneumonia, to a severe form known as Weil’s disease with symptoms that may include jaundice, bleeding tendency, renal failure, and pulmonary hemorrhage [1–3]. Because of the wide range of manifestations, diagnosis is often difficult, particularly in the absence of diagnostic resources, often leading to underreporting and even neglect [4].

Earlier studies have suggested that rainfall and floods might be important risk factors for leptospirosis in humans [7, 8]. An association between rainfall and leptospirosis has been reported in Sri Lanka, Reunion Island and Thailand [9–11]. Additionally, outbreaks of leptospirosis have been observed after floods in India, Philippines, Nicaragua, and other countries [12–16]. In the Philippines, given its location in the tropics along the typhoon belt, leptospirosis is endemic and outbreaks tend to occur after flooding or heavy rainfall [12, 17]. A recent study using data from this location has indicated correlations between the seasonal cycle of temperature, rainfall and humidity and the disease, but without controlling for potential mutual confounding [18]. In addition, no study has so far considered the non-linear or lagged associations between rainfall and leptospirosis, which are likely to be important due to qualitatively different mechanisms of disease transmission under light, heavy, or extreme rainfall, as well as the many sources of variability in the temporal lag between exposures and leptospirosis. Against this backdrop, in the current study, we used distributed lag non-linear model (DLNM)–a method that allows simultaneous representation of non-linear exposure-response relationships and delayed effects–to examine the short-term association between rainfall and leptospirosis, and the potential influence of floods in an urban setting.

## Methods

### Ethics statement

This study utilized secondary data. No human or animal subjects were used.

### Study area

Metro Manila is the capital of the Philippines covering an area of about 614km^2^ with an estimated population of 13 million [19]. Approximately a third of the population are living in slums, where many are at high risk for various infectious diseases because of poor sanitation [20]. The city has a tropical monsoon climate characterized by heavy rains from June to November, and recorded about 20 typhoons, cyclones or tropical depressions per year on average. The wet climate and the flat and low-lying geography of the city explain the frequent flooding, further made worse by poor drainage and waste management [21].

### Case data

Data on leptospirosis cases were obtained from the San Lazaro Hospital (SLH) admission database. SLH is a national referral hospital for infectious diseases in Manila. It has 500 beds and accepts a large number of patients from low social-economic background from the city and the surrounding regions. Leptospirosis is usually diagnosed clinically at SLH without serological confirmation because of limited diagnostic resources. A recent study at the same hospital suggests that the clinical diagnosis agreed with serological diagnoses in 63% of the cases [22]. Among the patients admitted during the period from 1 January 2001 to 31 December 2012, 3,590 were diagnosed with leptospirosis by physicians. Among these, 3,078 patients who were residing in Metro Manila were enrolled in the current study. We collected the demographic (age and sex) and clinical information (date of admission, final diagnosis, and outcome), and classified the patients into two age groups: “child” (0–15 years old) and “adult” (over 15 years old). To maintain patient confidentiality, we deleted individual-identifiable information and assigned identification number to each enrolled case. This study was approved by the ethical committee of SLH.

### Meteorological and flood data

Meteorological data were obtained from an online database [23]. We extracted daily rainfall (mm) and mean temperature (°C) for the study period from a meteorological station in Manila city about 3km south of the hospital (S1 Fig). Flood information was obtained from an online database of the Centre for Research on the Epidemiology of Disasters (CRED) at the Catholic University of Louvain, Belgium [24]. We collected data on “flood” and “storm” that occurred in Metro Manila from 2001 to 2012. This database includes only major events that fulfill at least one of the following criteria: (1) 10 or more people killed; (2) 100 or more people affected; (3) significant disaster; and/or (4) declaration of a state of emergency or an appeal for international assistance. The CRED database classifies disasters according to the nature of the primary event. For example, if a major storm was followed by flooding, the event is recorded in the database with the identifier 'storm'. Therefore, we collected data on 'flood' and 'storm'. These conditions exclude minor floods, which were therefore not considered in our analysis. For each event, we identified the start and end dates and created a binary variable to indicate the presence of a flood or storm on at least one day in a week.

### Pathways of infection

**Fig 1:**
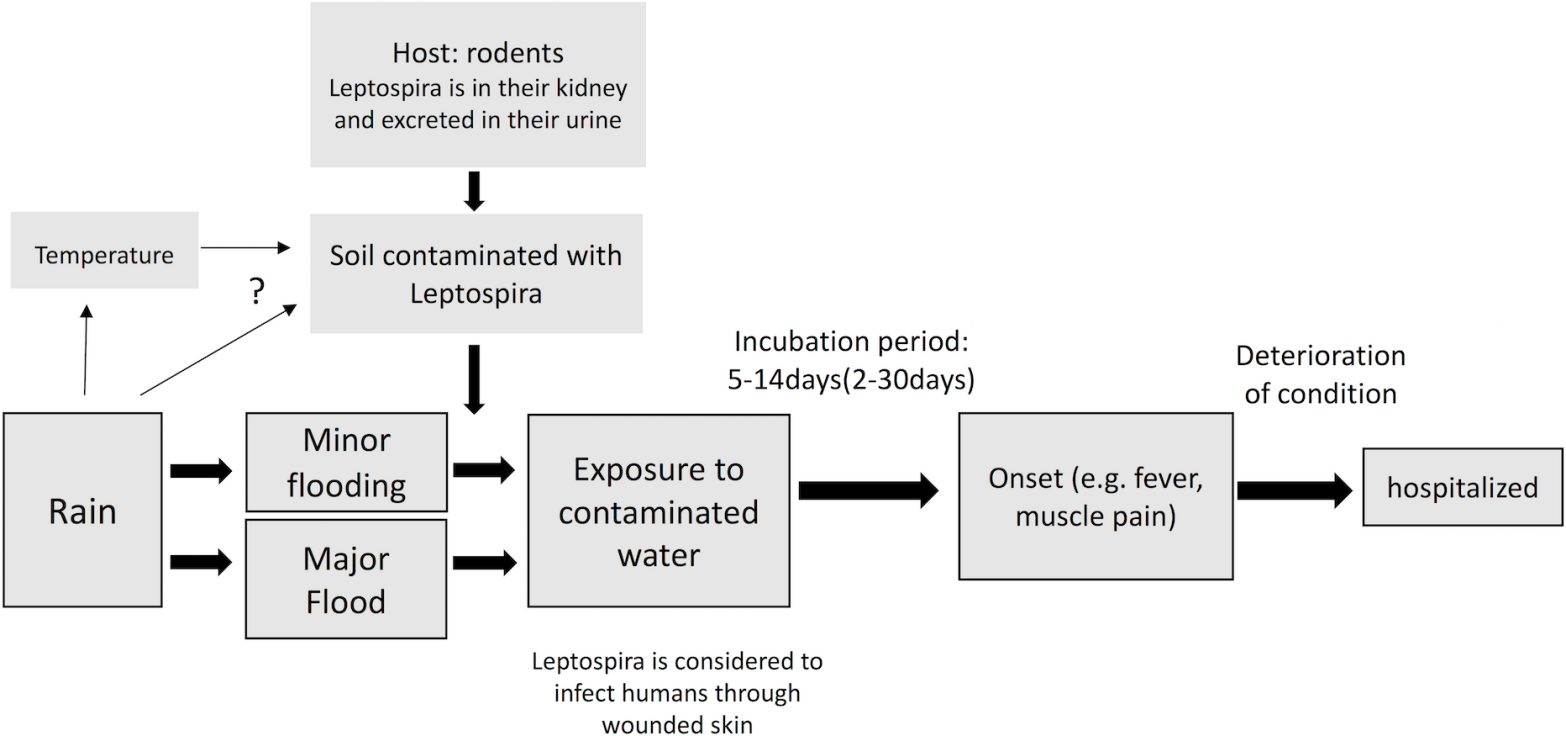
Assumed causal relationships among rainfall, flood and leptospirosis and other environmental factors.

The relationships between rainfall, flooding and leptospirosis hypothesized in this study are shown in Fig 1. Briefly, humans can become infected with *Leptospira* through exposure to contaminated water during floods. Flooding can also force humans and rodents into closer contact, which can result in further contamination of surrounding water. Although rodent populations may possibly affect the associations between rainfall and leptospirosis, we were unable to account for this interaction because we had no information about rodents [1, 3].

### Statistical analysis

We performed a time-series regression analysis by applying a DLNM in a quasi-Poisson regression framework in order to concurrently describe the non-linear relationship between rainfall and weekly hospital admissions for leptospirosis over multiple weeks of lag [25]. We allowed lags up to 4 weeks (lags 0–4) for rainfall, which were sufficient to capture attenuation of the association based on initial analysis (S2 Fig). For flood indicator, lags up to 7 weeks (lags 0–7) were included using a similar approach. We included weekly means of temperature averaged over lags 1–3 to adjust for the variable. The choice of lag period was based on previous studies [10, 11] and the understanding of the underlying mechanism linking *Leptospira* to human infection [1–3]. To control for seasonality and long-term trends in leptospirosis incidence due to effects other than the short-term impacts of rainfall, flooding, or temperature, we incorporated two natural cubic splines: one for the week of year with 3 degrees of freedom (DF) and another for year with 2 DF. The selection of DF was guided by the quasi-Akaike’s Information Criterion [26]. In the case of the rainfall, flood and temperature variables, we also considered the attenuation of the lag structure. The model has the following form:

$$log\left[E\left(Y_{t}\right)\right]=\alpha +\beta_{1}R_{t}{}_{,l}+\beta_{2}NCS\left(temperature,\;df=2\right)+\beta_{3}NCS\left(weekoy,\;df=3\right)+\beta_{4}NCS\left(year,\;df=2\right)$$

where Y_*t*_ is the number of leptospirosis cases in week *t*, α is the intercept, R_*t*,*l*_ is the matrix obtained by applying cross-basis function to rainfall through DLNM using natural cubic spline functions for rainfall and its lags, both at 3 DF, *l* is the lag of week, NCS is a natural cubic spline, “temperature”, “weekoy” and “year” denote the weekly mean temperature, week of year, and year, respectively. To account for the potential effect of floods, this model was expanded to include a cross-basis function of flood with strata parameterization for the variable space and natural cubic spline for the lag space with 5 DF. The collinearity between this indicator and rainfall was examined by checking their variance inflation factors (VIF).

We computed the relative risks (RRs) for rainfall at each lag using 0 cm/week rainfall as the reference. The RRs were expressed according to the flood warning system by the meteorological agency of the Philippines, PAGASA [27]. The system classifies the amount of rainfall into five categories–light, moderate, heavy, intense, and torrential–correspond to about 2–5, 5–15, 16–31, 32–63, and >63 cm/week of rainfall, respectively, with the assumption that rainfalls continue for 3 hours per day for 7 days. We presented the RRs at 2, 5, 16, 32, and 63 cm/week rainfall cut-off points. We also performed subgroup analyses by age and sex using the flood-unadjusted model.

**Fig 2:**
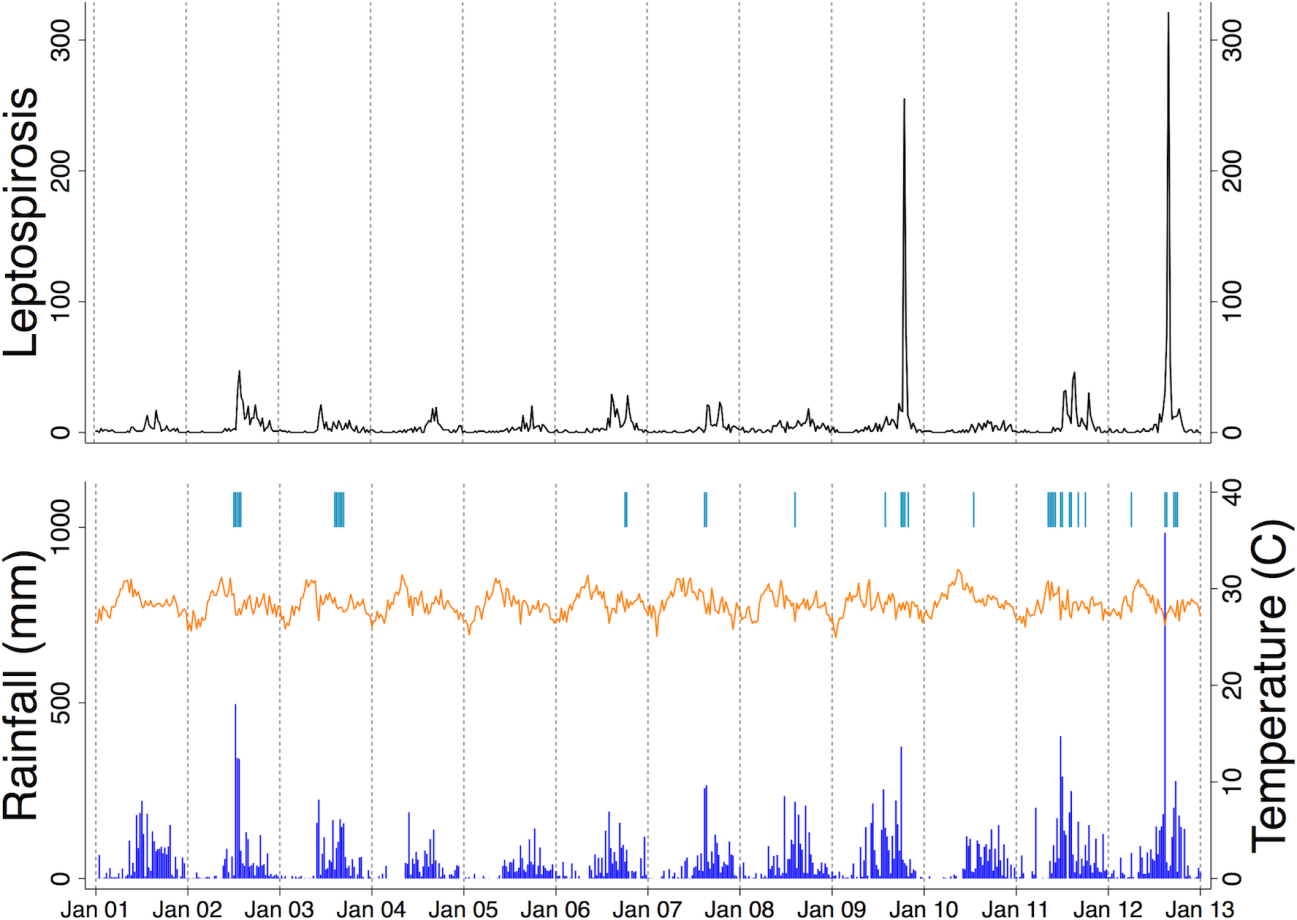
Weekly number of admitted leptospirosis cases, cumulative rainfall, mean temperature and flood events. (upper panel) Time series of the number of leptospirosis cases per week admitted to San Lazaro Hospital. (lower panel) Weekly cumulative rainfall (mm) (vertical bars), weekly mean temperature (°C) (solid line) and flood events (tick marks at the top).

We assessed the sensitivity of our results by changing the DF (3 to 7) for the week-of-year spline. We repeated the analysis to exclude extreme events using two subsets of data as follows. Subset 1 in which extreme rainfall in the 32^nd^ week of 2012 was removed, and Subset 2 in which, in addition to removing extreme rainfall in the 32^nd^ week of 2012, as in Subset 1, two outbreaks in the 41^st^ week of 2009 and the 34^th^ week of 2012 were removed (Fig 2). The weekly outcomes corresponding to these extreme values were regarded as missing during the sensitivity analysis. The exclusion of extreme rainfall resulted in no observation with >63 cm/week of rainfall and so no results are reported for this category.

Analysis was conducted using Stata version 12 (StataCorp LP, College Station, TX, USA) and R version 3.3.1 (R Foundation for Statistical Computing, Vienna, Austria). We used R packages *dlnm* (version 2.2.6) and *tsModel* (version 0.6).

## Results

There were 3,078 cases of leptospirosis admitted to SLH from January 2001 to December 2012 (Table 1). During this period, 253 of these patients died, translating to a case fatality rate of 8.4%. As observed in previous studies, leptospirosis was more frequently reported among adults (88.8%) and males (89.2%) [4, 28–30]. The weekly number of admitted leptospirosis cases, cumulative rainfall and mean temperature are shown in Fig 2 (The summary statistics were shown in S1 Table). Leptospirosis admissions displayed apparent seasonality with higher number of cases during the rainy season from June to November. Huge outbreaks were documented in 2009 and 2012. These outbreaks were preceded by heavy rainfalls, particularly in 2012 when an extreme level of rainfall was recorded. Mean temperature did not vary much, except for a slight increase around April and May.

**Table 1:** Summary statistics of the weekly number of leptospirosis cases admitted to San Lazaro Hospital in Manila, the Philippines in 2001–2012.

Characteristic		Cases	Mean	SD	Min	Max
Total		3,078	4.9	17.8	0	321
Sex	Male	2,745	4.4	15.9	0	283
	Female	333	0.5	2.1	0	38
Age group	0-15yrs	346	0.6	2.4	0	50
	> 15yrs	2,732	4.4	15.7	0	271

**Fig 3:**
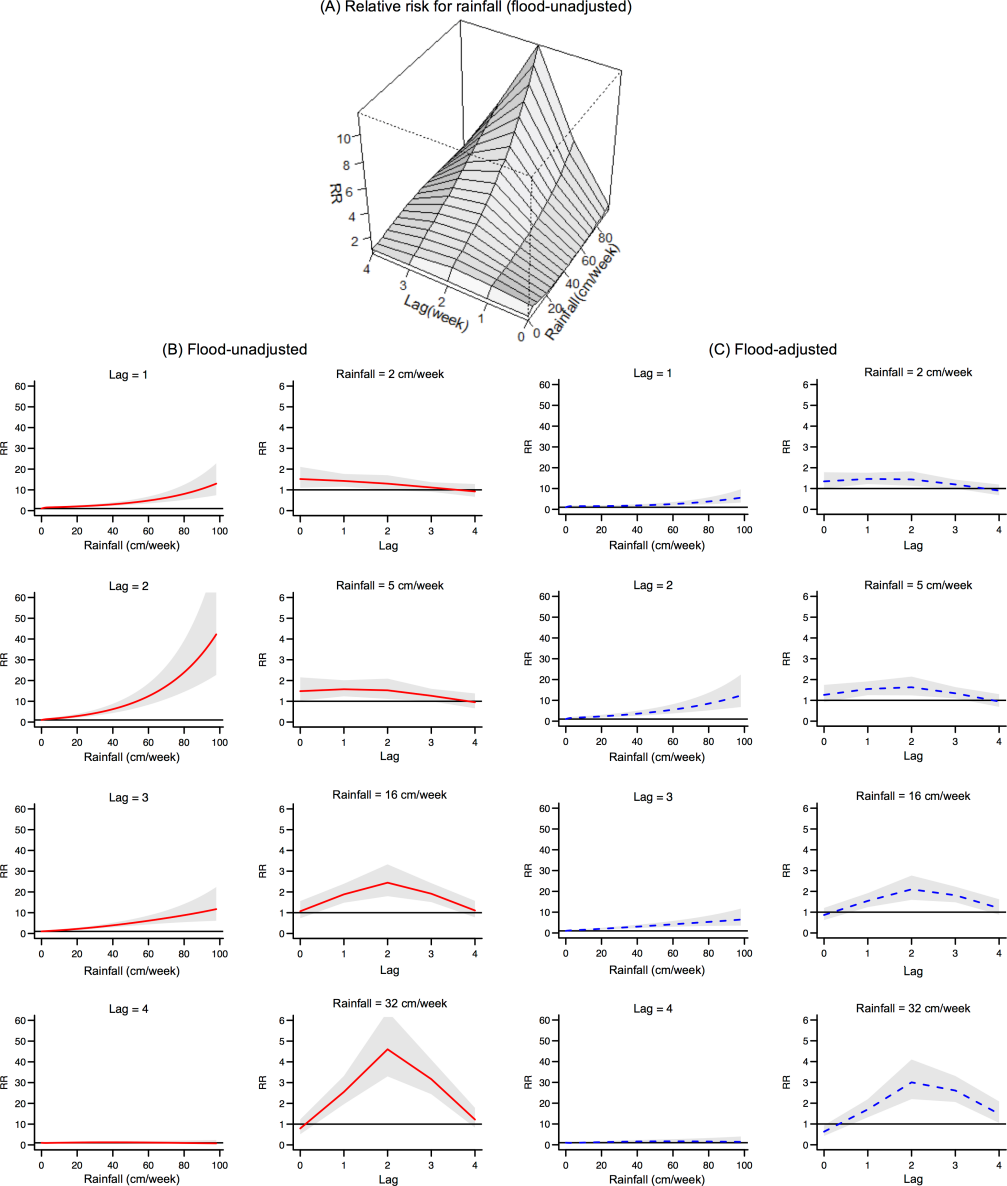
Lagged relationships between rainfall and leptospirosis. (A) Mean fitted relative risk surface over lag and weekly rainfall (flood-unadjusted model), (B) Cross-sectional plots of Fig 3(A) at constant lag / rainfall values with relative risk (RR) (solid red line) and 95% CIs (gray area) and (C) Cross-sectional plots at constant lag / rainfall values (flood-adjusted model) with relative risk (RR) (dotted blue line) and 95% CIs (gray area).

The estimated association between leptospirosis and rainfall, and the lagged pattern are shown in Fig 3. The risk of admission increased as the amount of rainfall increased, and this occurred mainly at lag 2 (Fig 3A). The cross-sectional plots by lag (Fig 3B, left panels) show a non-linear positive association between increasing rainfall and leptospirosis hospitalizations, with the strongest effects observed for heavy to torrential rainfall at a 2-week lag (Fig 3B, right panels). When adjusted for flood occurrence, RRs for rainfall of 16cm/week and 32cm/week at lag 2 decreased, but the lag structure was stable (Fig 3C).

The estimated RRs for the association are shown in S2 Table. The RRs of light, moderate, heavy, intense and torrential rainfall categories at a lag of 2 weeks were 1.30 (95% CI: 0.99–1.70), 1.53 (95% CI: 1.12–2.09), 2.45 (95% CI: 1.80–3.33), 4.61 (95% CI: 3.30–6.43), and 13.77 (95% CI: 9.10–20.82), respectively, compared with the reference of no rainfall (0 cm/week). The RRs were lower at lags 1 and 3 but significant at higher rainfall categories (S2 Table). There was no evidence of association at any rainfall category at lag 4.

When adjusted for flood occurrence, the RRs at lag 2 reduced for rainfall levels categorized as heavy, intense and torrential (S3 Table). The RRs for heavy, intense and torrential rainfall at lag 2 decreased by 14.3%, 34.9%, and 57.3%, respectively. The lag pattern was similar to that obtained with the flood-unadjusted model (S3 Table).

In the subgroup analysis, RRs at lag 2 were higher in the child groups for all rainfall categories but the difference was not significant. The lag patterns were similar among the subgroups (S4 Table and S5 Table).

**Fig 4:**
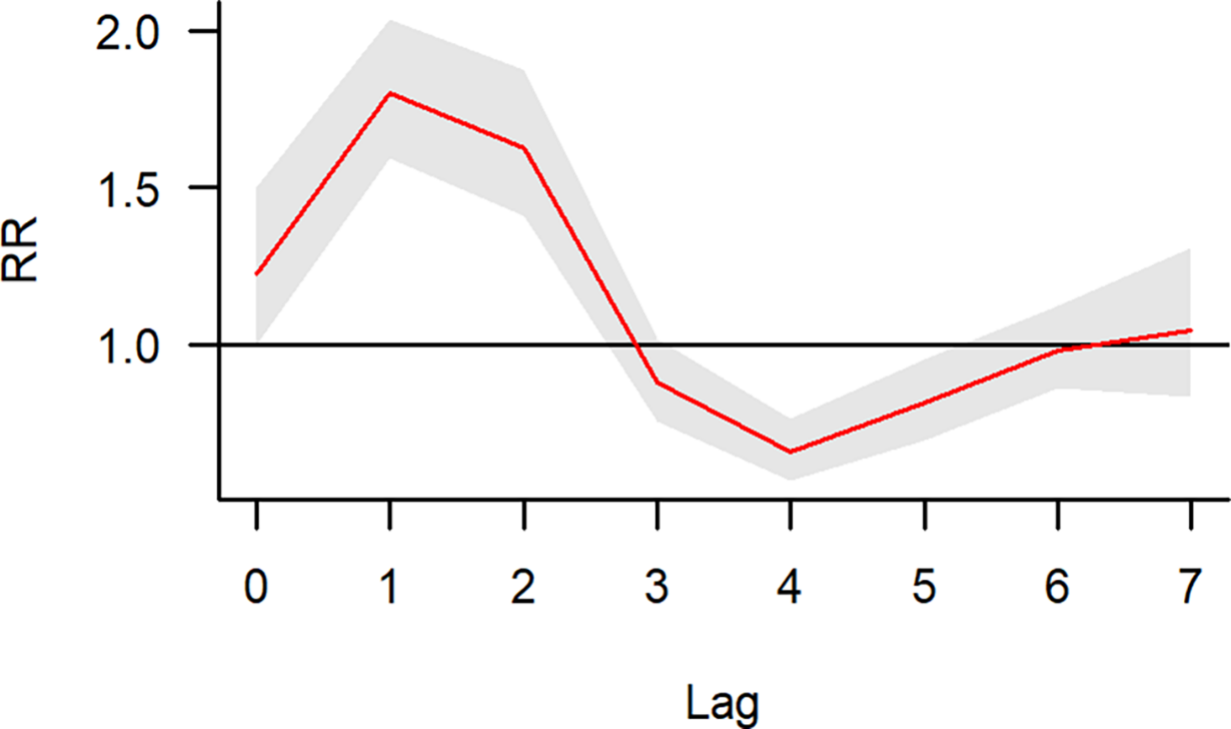
The relationship between flood occurrence and leptospirosis at lags 0 to 7 weeks.

Positive associations of flooding with leptospirosis were observed at lags of 1–2 weeks. The associations became negative at lag 4 and 5, while no association was observed at lags 6 and 7. The lag structure remained similar after removing extreme values. (Fig 4 and S6 Table). The VIFs for the flood indicator and rainfall cross-basis were less than 2.

In sensitivity analyses, the RRs associated with each level of rainfall remained stable after a week of extreme rainfall was removed from the dataset (Subset 1), and, with the exception of RRs associated with the ‘intense’ rainfall category, after the two most extreme weeks of outcome data were also removed (Subset 2) (S2 Table, S3 Table and S3 Fig). The RRs for all rainfall levels were not sensitive to the DF of the week-of-year spline (S7 Table) and rainfall remained a significant risk factor at lag 2 in all cases.

## Discussion

In this study, we investigated the short-term association between rainfall and leptospirosis. We found positive non-linear associations between rainfall and leptospirosis, with the strongest associations observed at a lag of 2 weeks. After adjusting for flooding, this association weakened, suggesting that floods might partially explain the effect of high rainfall on leptospirosis.

The time-series regression analysis suggested a significant positive association between the number of weekly admitted leptospirosis cases and the total weekly rainfall, with the greatest risk occurring 2 weeks after a heavy, intense, or torrential rainfall. This time lag is consistent with reports of leptospirosis outbreaks after floods. According to studies in India and Hawaii, leptospirosis outbreaks occurred 2 to 3 weeks after a flood [14, 16, 31]. The lag of 2 weeks might reflect the period from contaminated flood water exposure to hospital admission, which spans the incubation period as well as the onset and deterioration of the patient’s condition. At lower rainfall levels, the observed association between rainfall and leptospirosis at lags shorter than 2 weeks might be explained by rainfalls that gradually tapered off over a few weeks after an extreme event.

The association of flood with leptospirosis admissions was positive at lag week 1 and 2, but became negative at lag week 4 and 5, and null thereafter. This pattern resembles the so-called harvesting [32, 33], a term used to describe the forward displacement of health events such as leptospirosis admissions following an environmental trigger such as major flood. The increased risk observed at lag week 1 and 2 suggests that major floods have led to many admissions cases, resulting in a temporary reduction of susceptible population at lag week 4 and 5, which would explain the negative associations. However, its mechanism in the context of leptospirosis is not well understood. The observed lag pattern might be due to harvesting as explained, or other mechanisms that might produce similar harvesting-like time-series dynamics which would require further investigations.

Huge outbreaks of leptospirosis occurred in 2009 and 2012, both at times shortly after heavy rainfall and flooding were reported. The epidemic curve of leptospirosis was very steep, which suggests that many people were infected with the pathogen almost simultaneously or in a very short time, probably due to contact with flood water.

This study did not find association between rainfall and leptospirosis at a lag of 4 weeks or longer, although some previous studies have reported associations at lags lasting several months [9–11]. Studies in Sri Lanka and Reunion Island showed a 2-month lag in rainfall-case association in some parts of the study area [10, 11]. A study in the north and northeast Thailand showed 8 to 9-month lags in rainfall-case association in some parts of the study area [9]. It is unclear exactly why the results of the present study are different from these previous studies; however, differences in the geographic and demographic characteristics of the study sites may have contributed to this disparity. The previous studies were conducted in relatively rural areas, while this study was conducted in an urban area. The different conditions of the study area may have a different effect on the transmission of leptospirosis. In rural areas, farmers are exposed through their daily work to soil and water that is potentially contaminated with *Leptospira*. An increase in rainfall may make the soil moister, which helps *Leptospira* survive for a longer period of time. This lengthened lifespan could lead to an increased exposure to the bacteria among rodents, which might result in a higher number of *Leptospira*-infected rodents. Such situations would gradually increase occupational exposure to *Leptospira* among humans working in paddy fields in Sri Lanka or Thailand, or in sugar cane fields in Reunion Island. Through these processes, a corresponding increase in the number of leptospirosis cases could take several months from the observed rainfall. In contrast, in urban areas, most of the cases are likely infected by direct exposure to flood water. In such situations, the time from rainfall to a corresponding increase in the number of leptospirosis cases might only be slightly longer than the incubation time of leptospirosis.

The finding that the rainfall effects decreased after adjusting for flood at high rainfall level (> 16cm/week) suggests that flood is on the causal pathway between rainfall and the incidence of leptospirosis. Flood may increase the risk of leptospirosis because it increases the chance for exposure due to forced movement outside for evacuation and injuries to the skin. However, our flood indicator covers only relatively large-scale flood events, and smaller-scale floods were not considered in the analysis. The residual risks associated with high rainfall after adjusting for extreme floods could, in part, have to do with the role of smaller flood events. This is probably because people often wade in flooded streets in the rainy season in Manila.

There are several limitations to this study. First, the accuracy of the diagnosis for leptospirosis is uncertain because almost all cases of leptospirosis were diagnosed clinically in reference to the modified Faine’s diagnostic criteria [34, 35]. The criteria ask for history of contact with animal or contaminated water, which might possibly result in over diagnosis after flood. Laboratory tests such as IgM-ELISAs and MATs were seldom performed, and consistent serologically confirmed time series data were not available for analysis. A recent study showed that the clinical diagnoses of leptospirosis in SLH were consistent with serological diagnoses in 63% of the cases [22], suggesting possible over-diagnosis which is an important consideration when interpreting the results of the current study. Second, we did not take period from onset to hospitalization into account because we could not obtain information about date of onset. Leptospirosis has two-phase manifestations: early-phase manifestations with febrile illness which does not deteriorate shortly after the onset; severe late-phase manifestations with jaundice, renal failure, bleeding tendency and pulmonary haemorrhage which usually occur 4 to 6 days after the onset of illness. Although hospitalization occurs in late phase in general [3], the period from disease onset to hospital visit may vary. Third, this is a hospital-based study that included only admitted cases, which do not represent the population of all leptospirosis patients in the study area. However, this is unlikely to alter the observed association in the current study because of the similar pathways of infection. Fourth, population immunity to leptospirosis epidemics is uncertain, and immunity to leptospirosis is strongly restricted to homologous serovars or closely related serovars of *Leptospira*. In a huge leptospirosis outbreak, if some percentage of the population were infected with *Leptospira* but were asymptomatic or experienced only mild cases, an outbreak of the same *Leptospira* serovar may not occur again for several years. We did not take immunity effect into account in this study [36, 37]. Finally, although rodent populations might possibly affect the associations between rainfall and leptospirosis, we were unable to account for this interaction because we had no information about rodents.

Improved understanding of environmental factors associated with leptospirosis may help to improve the ability to predict outbreaks, and contribute to preparing hospitals and clinics for increased number of patients in case of an outbreak. In addition, identification of the specific environmental factors underlying the disease incidence is a critical step towards understanding how global climate change might affect patterns of leptospirosis.

## Conclusions

This study found that high rainfall in Manila was strongly associated with increased leptospirosis hospitalizations two weeks later, while controlling for confounding by seasonality and between-year variation in leptospirosis admissions, as well as effects of temperature. The association between rainfall and leptospirosis admissions could be explained, in part, by major floods.

## Supporting information

S1 Table Summary statistics of exposure variables. (DOCX)

S2 Table Relationships between rainfall and leptospirosis using the flood-unadjusted model. The relationships were determined at lags 0 to 4 weeks according to different rainfall levels based on the flood warning system in the Philippines.
(DOCX)

S3 Table Relationships between rainfall and leptospirosis using the flood-adjusted model. The relationships were determined at lags 0 to 4 weeks according to different rainfall levels based on the flood warning system in the Philippines.
(DOCX)

S4 Table Relationships between rainfall and leptospirosis by sex and age groups using the flood-unadjusted model. The relationships were determined at lags of 0 to 4 weeks according to different rainfall levels based on the flood warning system in the Philippines.
(DOCX)

S5 Table Relationships between rainfall and leptospirosis by sex and age groups using the flood-adjusted model. The relationships were determined at lags 0 to 4 weeks according to different rainfall levels based on the flood warning system in the Philippines.
(DOCX)

S6 Table Relationships between flood and leptospirosis at lags 0 to 7 weeks. (DOCX)

S7 Table Relative risks (RRs) for the association between rainfall and leptospirosis at a lag of 2 weeks using different degrees of freedom for the week-of-year spline for adjustment. (DOCX)

S1 Fig Map of Metro Manila, the Philippines, showing San Lazaro Hospital and the weather observatory. (DOCX)

S2 Fig Lagged relationships between rainfall and leptospirosis with the estimated relative risks (RRs) (solid red line) and 95% CI (gray area) over lags of 0 to7 weeks. Each graph shows the relationship at lags 1 to 7 weeks for rainfall at 5, 16, 32 and 63 cm/week with 0cm/week rainfall as the reference. Based on this result, lags 0 to 4 weeks were selected for the final model.
(DOCX)

S3 Fig Relationships between rainfall and leptospirosis at the rainfall level of 32cm/week (right panel) and at lag of 2 weeks (left panel). (A) Subset 1, flood-unadjusted model (B) Subset 1, flood-adjusted model (C) Subset 2, flood-unadjusted model (D) Subset 2, flood-adjusted model. Refer to the caption of S1 Table for descriptions of these datasets.
(DOCX)

S1 Dataset All data without personally identifiable information are provided. (XLSX)
