# Supplementary material for: The non-linear and lagged short-term relationship between rainfall and leptospirosis and the intermediate role of floods in the Philippines
Source: PLoS Negl Trop Dis. 2018 Apr 16;12(4):e0006331. doi: 10.1371/journal.pntd.0006331 (PMC5919665; doi:10.1371/journal.pntd.0006331)
Supplement: S1 Table — (DOCX) [file pntd.0006331.s001.docx]

**S1 Table.** Summary statistics of the exposure variables

|  | Mean | SD | Min | 25th percentile | Median | 75th percentile | Max |
| --- | --- | --- | --- | --- | --- | --- | --- |
| Rainfall (cm/week) | 4.48 | 7.28 | 0.00 | 0.18 | 1.90 | 5.80 | 98.40 |
| Flood YES (39weeks) | 17.13 | 7.28 | 0.10 | 4.72 | 10.47 | 25.32 | 98.40 |
| Flood NO (585weeks) | 3.63 | 18.41 | 0.00 | 0.15 | 1.63 | 5.20 | 25.40 |
| Temperature(℃) | 28.44 | 1.19 | 24.97 | 27.67 | 28.37 | 29.18 | 31.97 |
