# Supplementary material for: The non-linear and lagged short-term relationship between rainfall and leptospirosis and the intermediate role of floods in the Philippines
Source: PLoS Negl Trop Dis. 2018 Apr 16;12(4):e0006331. doi: 10.1371/journal.pntd.0006331 (PMC5919665; doi:10.1371/journal.pntd.0006331)
Supplement: S2 Table — The relationships were determined at lags 0 to 4 weeks according to different rainfall levels based on the flood warning system in the Philippines. (DOCX) [file pntd.0006331.s002.docx]

**S2 Table.** Relationships between rainfall and leptospirosis using the flood-unadjusted model. The relationships were determined at lags 0 to 4 weeks according to different rainfall levels based on the flood warning system in the Philippines.

|  |  | Lag 0 | | | Lag 1 | | | Lag 2 | | | Lag 3 | | | Lag 4 | | |
| --- | --- | --- | --- | --- | --- | --- | --- | --- | --- | --- | --- | --- | --- | --- | --- | --- |
| Rainfall Level | Dataset | RR | 95% CI | | RR | 95% CI | | RR | 95% CI | | RR | 95% CI | | RR | 95% CI | |
|  |  |  |  |  |  |  |  |  |  |  |  |  |  |  |  |  |
| Light rain | Full data | 1.52 | 1.10 | 2.11 | 1.43 | 1.16 | 1.77 | 1.30 | 0.99 | 1.70 | 1.11 | 0.91 | 1.37 | 0.92 | 0.67 | 1.28 |
| (2cm/week) | Subset 1 | 1.43 | 1.05 | 1.94 | 1.55 | 1.28 | 1.89 | 1.50 | 1.16 | 1.93 | 1.19 | 0.98 | 1.44 | 0.84 | 0.62 | 1.13 |
|  | Subset 2 | 1.20 | 0.93 | 1.56 | 1.38 | 1.17 | 1.63 | 1.42 | 1.14 | 1.76 | 1.21 | 1.03 | 1.42 | 0.92 | 0.71 | 1.18 |
|  |  |  |  |  |  |  |  |  |  |  |  |  |  |  |  |  |
| Moderate rain | Full data | 1.49 | 1.03 | 2.15 | 1.58 | 1.24 | 2.01 | 1.53 | 1.12 | 2.09 | 1.27 | 1.00 | 1.60 | 0.95 | 0.66 | 1.38 |
| (5cm/week) | Subset 1 | 1.33 | 0.95 | 1.87 | 1.56 | 1.25 | 1.94 | 1.60 | 1.20 | 2.13 | 1.33 | 1.07 | 1.64 | 0.97 | 0.70 | 1.34 |
|  | Subset 2 | 1.14 | 0.85 | 1.51 | 1.42 | 1.18 | 1.70 | 1.52 | 1.20 | 1.94 | 1.29 | 1.08 | 1.55 | 0.94 | 0.71 | 1.25 |
|  |  |  |  |  |  |  |  |  |  |  |  |  |  |  |  |  |
| Heavy rain | Full data | 1.07 | 0.74 | 1.56 | 1.88 | 1.48 | 2.39 | 2.45 | 1.80 | 3.33 | 1.92 | 1.51 | 2.42 | 1.11 | 0.78 | 1.57 |
| (16cm/week) | Subset 1 | 0.83 | 0.58 | 1.18 | 1.46 | 1.15 | 1.85 | 2.02 | 1.51 | 2.72 | 1.88 | 1.50 | 2.34 | 1.36 | 0.99 | 1.87 |
|  | Subset 2 | 0.88 | 0.66 | 1.19 | 1.43 | 1.17 | 1.75 | 1.82 | 1.42 | 2.34 | 1.55 | 1.28 | 1.88 | 1.04 | 0.79 | 1.37 |
|  |  |  |  |  |  |  |  |  |  |  |  |  |  |  |  |  |
| Intense rain | Full data | 0.79 | 0.51 | 1.23 | 2.55 | 1.96 | 3.32 | 4.61 | 3.30 | 6.43 | 3.17 | 2.44 | 4.10 | 1.22 | 0.84 | 1.78 |
| (32cm/week) | Subset 1 | 0.49 | 0.30 | 0.80 | 2.31 | 1.78 | 2.99 | 5.13 | 3.78 | 6.97 | 3.21 | 2.53 | 4.07 | 0.94 | 0.64 | 1.37 |
|  | Subset 2 | 0.79 | 0.53 | 1.17 | 1.99 | 1.61 | 2.47 | 3.14 | 2.40 | 4.11 | 2.25 | 1.83 | 2.77 | 1.01 | 0.74 | 1.37 |
|  |  |  |  |  |  |  |  |  |  |  |  |  |  |  |  |  |
| Torrential rain | Full data | 0.70 | 0.40 | 1.23 | 5.21 | 3.68 | 7.38 | 13.77 | 9.10 | 20.82 | 6.51 | 4.55 | 9.31 | 1.09 | 0.62 | 1.92 |
| (63cm/week) | Subset 1 | - | - | - | - | - | - | - | - | - | - | - | - | - | - | - |
|  | Subset 2 | - | - | - | - | - | - | - | - | - | - | - | - | - | - | - |

The reference of RRs was the risk at no rainfall.

RRs were estimated using 3 datasets (Full data, Subset 1 and Subset 2). Full data: all observations. Subset 1: One week of heavy rainfall (32^nd^ week in 2012) was excluded. Subset 2: One week of heavy rainfall (32^nd^ week in 2012) and two weeks of outbreaks (41^st^ week in 2009 and 34^th^ week in 2012) were excluded.
