# Supplementary material for: The non-linear and lagged short-term relationship between rainfall and leptospirosis and the intermediate role of floods in the Philippines
Source: PLoS Negl Trop Dis. 2018 Apr 16;12(4):e0006331. doi: 10.1371/journal.pntd.0006331 (PMC5919665; doi:10.1371/journal.pntd.0006331)
Supplement: S4 Table — The relationships were determined at lags of 0 to 4 weeks according to different rainfall levels based on the flood warning system in the Philippines. (DOCX) [file pntd.0006331.s004.docx]

**S4 Table.**　Relationships between rainfall and leptospirosis by sex and age groups using the flood-unadjusted model. The relationships were determined at lags of 0 to 4 weeks according to different rainfall levels based on the flood warning system in the Philippines.

|  |  | Lag 0 | | | Lag 1 | | | Lag 2 | | | Lag 3 | | | Lag 4 | | |
| --- | --- | --- | --- | --- | --- | --- | --- | --- | --- | --- | --- | --- | --- | --- | --- | --- |
|  | Rainfall Level | RR | 95% CI | | RR | 95% CI | | RR | 95% CI | | RR | 95% CI | | RR | 95% CI | |
| Male | Light | 1.50 | 1.08 | 2.08 | 1.40 | 1.13 | 1.73 | 1.27 | 0.97 | 1.68 | 1.11 | 0.90 | 1.37 | 0.95 | 0.68 | 1.32 |
|  | Moderate | 1.46 | 1.00 | 2.12 | 1.54 | 1.21 | 1.97 | 1.50 | 1.09 | 2.06 | 1.27 | 1.00 | 1.61 | 0.99 | 0.68 | 1.43 |
|  | Heavy | 1.04 | 0.71 | 1.53 | 1.84 | 1.44 | 2.34 | 2.41 | 1.76 | 3.29 | 1.93 | 1.52 | 2.45 | 1.15 | 0.80 | 1.64 |
|  | Intense | 0.76 | 0.49 | 1.18 | 2.49 | 1.90 | 3.25 | 4.56 | 3.25 | 6.40 | 3.19 | 2.45 | 4.15 | 1.25 | 0.85 | 1.84 |
|  | Torrential | 0.64 | 0.36 | 1.14 | 5.05 | 3.54 | 7.21 | 13.83 | 9.08 | 21.08 | 6.51 | 4.52 | 9.39 | 1.07 | 0.60 | 1.91 |
|  |  |  |  |  |  |  |  |  |  |  |  |  |  |  |  |  |
| Female | Light | 1.73 | 1.00 | 3.00 | 1.71 | 1.20 | 2.44 | 1.52 | 0.96 | 2.38 | 1.12 | 0.81 | 1.56 | 0.75 | 0.45 | 1.25 |
|  | Moderate | 1.75 | 0.94 | 3.28 | 1.95 | 1.30 | 2.93 | 1.82 | 1.08 | 3.06 | 1.26 | 0.86 | 1.85 | 0.74 | 0.41 | 1.31 |
|  | Heavy | 1.34 | 0.71 | 2.54 | 2.33 | 1.55 | 3.48 | 2.81 | 1.68 | 4.69 | 1.85 | 1.26 | 2.72 | 0.85 | 0.49 | 1.47 |
|  | Intense | 1.11 | 0.54 | 2.31 | 3.17 | 2.04 | 4.92 | 5.01 | 2.88 | 8.73 | 2.99 | 1.95 | 4.58 | 0.99 | 0.54 | 1.81 |
|  | Torrential | 1.29 | 0.55 | 3.05 | 6.55 | 3.72 | 11.51 | 13.52 | 6.83 | 26.78 | 6.26 | 3.53 | 11.11 | 1.18 | 0.53 | 2.66 |
|  |  |  |  |  |  |  |  |  |  |  |  |  |  |  |  |  |
| Adult | Light | 1.54 | 1.11 | 2.15 | 1.38 | 1.12 | 1.72 | 1.23 | 0.94 | 1.63 | 1.08 | 0.88 | 1.33 | 0.95 | 0.68 | 1.32 |
|  | Moderate | 1.50 | 1.03 | 2.19 | 1.53 | 1.19 | 1.95 | 1.45 | 1.06 | 1.99 | 1.23 | 0.97 | 1.56 | 0.98 | 0.67 | 1.43 |
|  | Heavy | 1.07 | 0.73 | 1.57 | 1.84 | 1.44 | 2.35 | 2.38 | 1.74 | 3.25 | 1.89 | 1.49 | 2.41 | 1.13 | 0.79 | 1.62 |
|  | Intense | 0.78 | 0.50 | 1.23 | 2.52 | 1.92 | 3.29 | 4.56 | 3.25 | 6.40 | 3.18 | 2.44 | 4.14 | 1.25 | 0.85 | 1.84 |
|  | Torrential | 0.70 | 0.39 | 1.25 | 5.12 | 3.58 | 7.32 | 13.53 | 8.85 | 20.68 | 6.56 | 4.54 | 9.49 | 1.15 | 0.64 | 2.06 |
|  |  |  |  |  |  |  |  |  |  |  |  |  |  |  |  |  |
| Child | Light | 1.35 | 0.77 | 2.36 | 2.07 | 1.38 | 3.10 | 2.31 | 1.36 | 3.93 | 1.53 | 1.04 | 2.26 | 0.74 | 0.43 | 1.29 |
|  | Moderate | 1.34 | 0.71 | 2.53 | 2.37 | 1.49 | 3.77 | 2.83 | 1.54 | 5.22 | 1.77 | 1.13 | 2.77 | 0.75 | 0.40 | 1.40 |
|  | Heavy | 1.08 | 0.57 | 2.04 | 2.57 | 1.62 | 4.06 | 3.72 | 2.05 | 6.72 | 2.35 | 1.51 | 3.65 | 0.91 | 0.50 | 1.62 |
|  | Intense | 0.87 | 0.42 | 1.77 | 3.24 | 1.99 | 5.27 | 6.01 | 3.24 | 11.17 | 3.46 | 2.17 | 5.51 | 0.99 | 0.53 | 1.82 |
|  | Torrential | 0.72 | 0.29 | 1.75 | 6.85 | 3.72 | 12.62 | 19.15 | 9.17 | 39.97 | 6.87 | 3.74 | 12.62 | 0.72 | 0.30 | 1.73 |

RRs were estimated using Full data

Rainfall category: Light (2cm/week), Moderate (5cm/week), Heavy (16cm/week), Intense (32cm/week), Torrential (63cm/week)
