# Supplementary material for: The non-linear and lagged short-term relationship between rainfall and leptospirosis and the intermediate role of floods in the Philippines
Source: PLoS Negl Trop Dis. 2018 Apr 16;12(4):e0006331. doi: 10.1371/journal.pntd.0006331 (PMC5919665; doi:10.1371/journal.pntd.0006331)
Supplement: S5 Table — The relationships were determined at lags 0 to 4 weeks according to different rainfall levels based on the flood warning system in the Philippines. (DOCX) [file pntd.0006331.s005.docx]

**S5 Table.**Relationships between rainfall and leptospirosis by sex and age groups using the flood-adjusted model. The relationships were determined at lags 0 to 4 weeks according to different rainfall levels based on the flood warning system in the Philippines.

|  |  | Lag 0 | | | Lag 1 | | | Lag 2 | | | Lag 3 | | | Lag 4 | | |
| --- | --- | --- | --- | --- | --- | --- | --- | --- | --- | --- | --- | --- | --- | --- | --- | --- |
|  | Rainfall Level | RR | 95% CI | | RR | 95% CI | | RR | 95% CI | | RR | 95% CI | | RR | 95% CI | |
|  |  |  |  |  |  |  |  |  |  |  |  |  |  |  |  |  |
| Male | Light | 1.32 | 0.99 | 1.76 | 1.44 | 1.19 | 1.74 | 1.42 | 1.11 | 1.81 | 1.20 | 1.00 | 1.43 | 0.91 | 0.68 | 1.22 |
|  | Moderate | 1.23 | 0.89 | 1.72 | 1.51 | 1.22 | 1.88 | 1.61 | 1.22 | 2.12 | 1.34 | 1.09 | 1.65 | 0.96 | 0.70 | 1.34 |
|  | Heavy | 0.83 | 0.59 | 1.17 | 1.50 | 1.21 | 1.88 | 2.07 | 1.57 | 2.74 | 1.82 | 1.48 | 2.25 | 1.22 | 0.89 | 1.68 |
|  | Intense | 0.59 | 0.40 | 0.89 | 1.65 | 1.28 | 2.14 | 2.99 | 2.17 | 4.11 | 2.64 | 2.07 | 3.37 | 1.52 | 1.07 | 2.16 |
|  | Torrential | 0.56 | 0.32 | 0.98 | 2.57 | 1.81 | 3.66 | 5.99 | 3.96 | 9.05 | 4.44 | 3.12 | 6.32 | 1.66 | 0.96 | 2.85 |
|  |  |  |  |  |  |  |  |  |  |  |  |  |  |  |  |  |
| Female | Light | 1.56 | 0.92 | 2.65 | 1.69 | 1.20 | 2.37 | 1.60 | 1.03 | 2.46 | 1.20 | 0.88 | 1.65 | 0.79 | 0.49 | 1.29 |
|  | Moderate | 1.54 | 0.85 | 2.81 | 1.85 | 1.25 | 2.74 | 1.84 | 1.11 | 3.03 | 1.33 | 0.93 | 1.92 | 0.80 | 0.46 | 1.39 |
|  | Heavy | 1.16 | 0.63 | 2.15 | 1.90 | 1.27 | 2.85 | 2.32 | 1.40 | 3.85 | 1.73 | 1.19 | 2.51 | 0.96 | 0.57 | 1.64 |
|  | Intense | 0.99 | 0.49 | 2.02 | 2.16 | 1.36 | 3.45 | 3.15 | 1.78 | 5.58 | 2.34 | 1.52 | 3.61 | 1.16 | 0.64 | 2.12 |
|  | Torrential | 1.40 | 0.58 | 3.42 | 3.46 | 1.85 | 6.47 | 5.21 | 2.50 | 10.86 | 3.44 | 1.87 | 6.34 | 1.39 | 0.59 | 3.25 |
|  |  |  |  |  |  |  |  |  |  |  |  |  |  |  |  |  |
| Adult | Light | 1.36 | 1.02 | 1.81 | 1.40 | 1.16 | 1.69 | 1.35 | 1.06 | 1.72 | 1.16 | 0.97 | 1.39 | 0.94 | 0.70 | 1.25 |
|  | Moderate | 1.27 | 0.92 | 1.77 | 1.48 | 1.19 | 1.83 | 1.52 | 1.16 | 2.01 | 1.30 | 1.06 | 1.59 | 0.99 | 0.71 | 1.36 |
|  | Heavy | 0.86 | 0.61 | 1.20 | 1.48 | 1.19 | 1.84 | 1.99 | 1.51 | 2.63 | 1.77 | 1.44 | 2.18 | 1.23 | 0.90 | 1.68 |
|  | Intense | 0.61 | 0.41 | 0.92 | 1.63 | 1.26 | 2.10 | 2.87 | 2.09 | 3.94 | 2.55 | 2.00 | 3.25 | 1.51 | 1.06 | 2.14 |
|  | Torrential | 0.60 | 0.35 | 1.05 | 2.49 | 1.75 | 3.55 | 5.47 | 3.62 | 8.26 | 4.17 | 2.93 | 5.93 | 1.68 | 0.98 | 2.88 |
|  |  |  |  |  |  |  |  |  |  |  |  |  |  |  |  |  |
| Child | Light | 1.19 | 0.68 | 2.10 | 2.27 | 1.51 | 3.40 | 2.78 | 1.65 | 4.70 | 1.66 | 1.13 | 2.43 | 0.64 | 0.37 | 1.10 |
|  | Moderate | 1.15 | 0.61 | 2.19 | 2.56 | 1.61 | 4.07 | 3.40 | 1.86 | 6.21 | 1.92 | 1.24 | 2.98 | 0.65 | 0.35 | 1.20 |
|  | Heavy | 0.93 | 0.49 | 1.77 | 2.48 | 1.56 | 3.95 | 3.88 | 2.14 | 7.02 | 2.46 | 1.60 | 3.78 | 0.91 | 0.51 | 1.62 |
|  | Intense | 0.80 | 0.38 | 1.69 | 2.79 | 1.67 | 4.68 | 5.22 | 2.74 | 9.97 | 3.47 | 2.15 | 5.59 | 1.24 | 0.66 | 2.33 |
|  | Torrential | 0.96 | 0.35 | 2.58 | 5.30 | 2.72 | 10.33 | 12.26 | 5.53 | 27.17 | 6.57 | 3.41 | 12.65 | 1.46 | 0.58 | 3.73 |

RRs were estimated using Full data.

Rainfall level: Light (2cm/week), Moderate (5cm/week), Heavy (16cm/week), Intense (32cm/week), Torrential (63cm/week)
