# Supplementary material for: The non-linear and lagged short-term relationship between rainfall and leptospirosis and the intermediate role of floods in the Philippines
Source: PLoS Negl Trop Dis. 2018 Apr 16;12(4):e0006331. doi: 10.1371/journal.pntd.0006331 (PMC5919665; doi:10.1371/journal.pntd.0006331)
Supplement: S6 Table — (DOCX) [file pntd.0006331.s006.docx]

**S6 Table.** Relationships between flood and leptospirosis at lags 0 to 7 weeks.

|  | Full data | | | Subset 1 | | | Subset 2 | | |
| --- | --- | --- | --- | --- | --- | --- | --- | --- | --- |
| Lag | RR | 95% CI | | RR | 95% CI | | RR | 95% CI | |
| 0 | 1.23 | 1.00 | 1.50 | 1.30 | 1.06 | 1.61 | 0.76 | 0.60 | 0.96 |
| 1 | 1.80 | 1.59 | 2.03 | 1.67 | 1.48 | 1.89 | 1.28 | 1.12 | 1.45 |
| 2 | 1.63 | 1.41 | 1.87 | 1.43 | 1.23 | 1.65 | 1.44 | 1.25 | 1.66 |
| 3 | 0.88 | 0.76 | 1.02 | 0.82 | 0.70 | 0.95 | 1.01 | 0.88 | 1.17 |
| 4 | 0.66 | 0.56 | 0.77 | 0.67 | 0.57 | 0.78 | 0.80 | 0.70 | 0.93 |
| 5 | 0.81 | 0.69 | 0.95 | 0.87 | 0.74 | 1.02 | 0.87 | 0.76 | 1.00 |
| 6 | 0.98 | 0.86 | 1.12 | 1.03 | 0.90 | 1.16 | 1.00 | 0.89 | 1.12 |
| 7 | 1.04 | 0.83 | 1.31 | 1.00 | 0.80 | 1.25 | 1.12 | 0.93 | 1.36 |

RRs were estimated using 3 datasets (Full data, Subset 1 and Subset 2).

Full data: all observations

Subset 1: A week of heavy rainfall (32nd week in 2012) was excluded

Subset 2: A week of heavy rainfall (32nd week in 2012) and two weeks of outbreaks (41st week in 2009 and 34th week in 2012) were excluded
