# Supplementary material for: The non-linear and lagged short-term relationship between rainfall and leptospirosis and the intermediate role of floods in the Philippines
Source: PLoS Negl Trop Dis. 2018 Apr 16;12(4):e0006331. doi: 10.1371/journal.pntd.0006331 (PMC5919665; doi:10.1371/journal.pntd.0006331)
Supplement: S7 Table — (DOCX) [file pntd.0006331.s007.docx]

**S7 Table.**Relative risks (RRs) for the association between rainfall and leptospirosis at a lag of 2 weeks using different degrees of freedom for the week-of-year spline for adjustment.

|  | Degree of Freedom | | | | | | | | | | | | | | |
| --- | --- | --- | --- | --- | --- | --- | --- | --- | --- | --- | --- | --- | --- | --- | --- |
| Rainfall Level | 3* |  |  | 4 |  |  | 5 |  |  | 6 |  |  | 7 |  |  |
|  | RR | 95%CI | | RR | 95%CI | | RR | 95%CI | | RR | 95%CI | | RR | 95%CI | |
| Full data: |  |  |  |  |  |  |  |  |  |  |  |  |  |  |  |
| Light | 1.30 | 0.99 | 1.70 | 1.34 | 1.02 | 1.75 | 1.19 | 0.91 | 1.56 | 1.22 | 0.94 | 1.60 | 1.20 | 0.92 | 1.58 |
| Moderate | 1.53 | 1.12 | 2.09 | 1.58 | 1.16 | 2.16 | 1.37 | 1.01 | 1.87 | 1.42 | 1.04 | 1.93 | 1.39 | 1.02 | 1.90 |
| Heavy | 2.45 | 1.80 | 3.33 | 2.55 | 1.87 | 3.47 | 2.15 | 1.58 | 2.92 | 2.23 | 1.64 | 3.03 | 2.20 | 1.61 | 2.99 |
| Intense | 4.61 | 3.30 | 6.43 | 4.84 | 3.47 | 6.76 | 4.01 | 2.88 | 5.59 | 4.15 | 2.97 | 5.79 | 4.11 | 2.94 | 5.75 |
| Torrential | 13.77 | 9.10 | 20.82 | 14.64 | 9.70 | 22.11 | 12.43 | 8.29 | 18.64 | 12.66 | 8.42 | 19.05 | 12.61 | 8.38 | 19.00 |
|  |  |  |  |  |  |  |  |  |  |  |  |  |  |  |  |
| Subset 2: |  |  |  |  |  |  |  |  |  |  |  |  |  |  |  |
| Light | 1.42 | 1.14 | 1.76 | 1.42 | 1.14 | 1.77 | 1.29 | 1.04 | 1.60 | 1.33 | 1.08 | 1.65 | 1.32 | 1.06 | 1.64 |
| Moderate | 1.52 | 1.20 | 1.94 | 1.52 | 1.19 | 1.94 | 1.36 | 1.07 | 1.73 | 1.40 | 1.10 | 1.78 | 1.38 | 1.08 | 1.76 |
| Heavy | 1.82 | 1.42 | 2.34 | 1.80 | 1.40 | 2.32 | 1.58 | 1.23 | 2.03 | 1.62 | 1.26 | 2.07 | 1.59 | 1.24 | 2.06 |
| Intense | 3.14 | 2.40 | 4.11 | 3.12 | 2.38 | 4.10 | 2.65 | 2.02 | 3.49 | 2.75 | 2.10 | 3.61 | 2.70 | 2.05 | 3.56 |

* Final model (full data)
