# Supplementary material for: The non-linear and lagged short-term relationship between rainfall and leptospirosis and the intermediate role of floods in the Philippines
Source: PLoS Negl Trop Dis. 2018 Apr 16;12(4):e0006331. doi: 10.1371/journal.pntd.0006331 (PMC5919665; doi:10.1371/journal.pntd.0006331)
Supplement: S1 Fig — (DOCX) [file pntd.0006331.s008.docx]

**
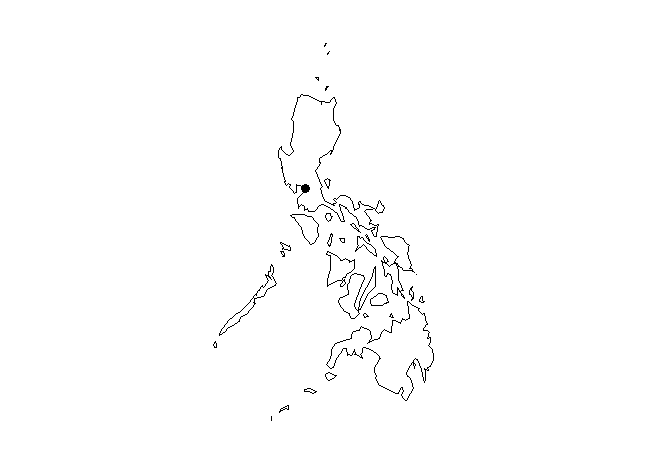
**


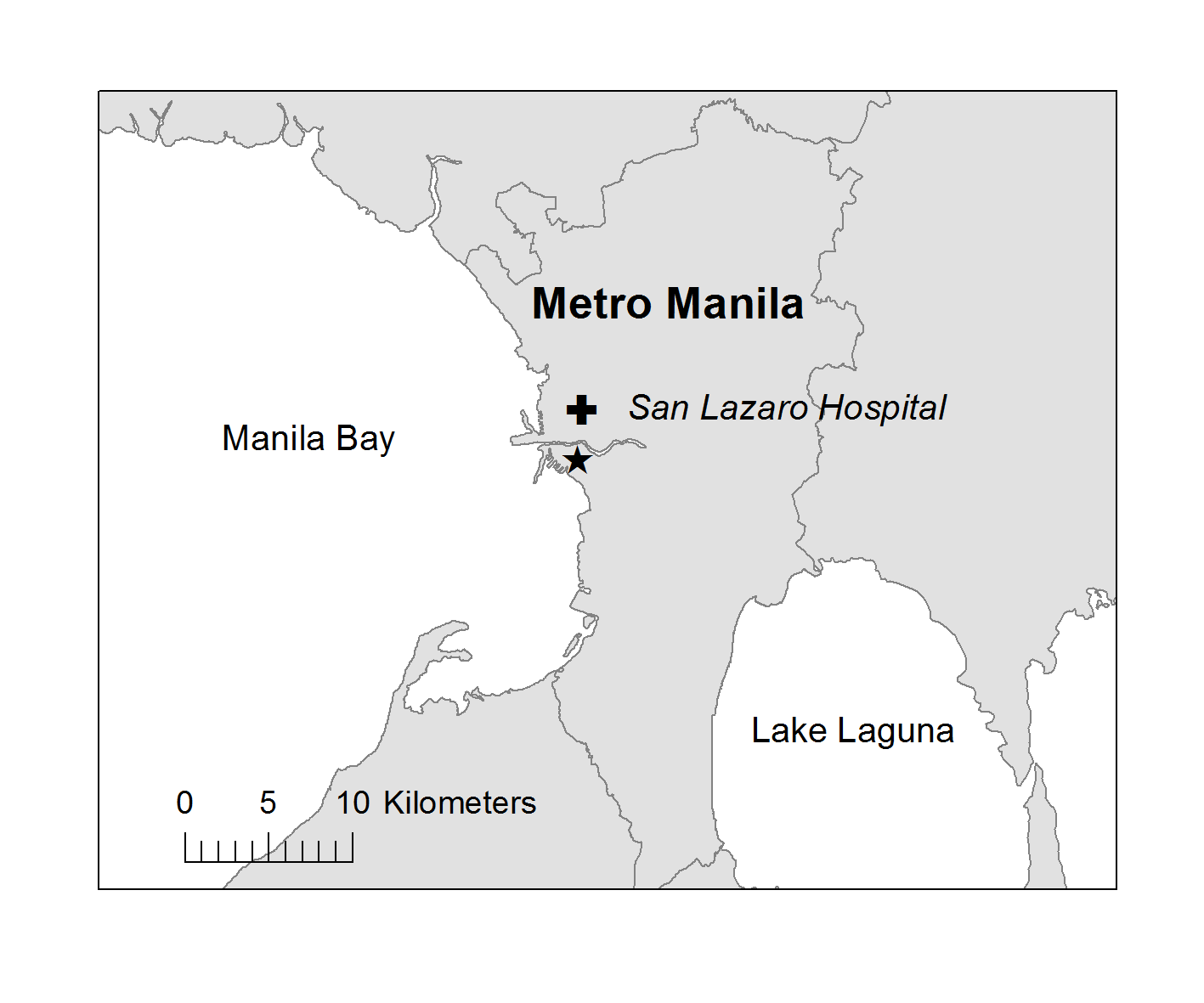


**S1 Fig.** Map of Metro Manila, the Philippines. San Lazaro Hospital (cross) and the weather observatory (star).
