# Supplementary material for: The non-linear and lagged short-term relationship between rainfall and leptospirosis and the intermediate role of floods in the Philippines
Source: PLoS Negl Trop Dis. 2018 Apr 16;12(4):e0006331. doi: 10.1371/journal.pntd.0006331 (PMC5919665; doi:10.1371/journal.pntd.0006331)

**S2 Fig.** Lagged relationships between rainfall and leptospirosis with the estimated RR (solid red line) and 95% CI (gray area) over lags 0-7. Each graph shows the relationship at lag week 1 to 7 for rainfall at 5, 16, 32 and 63 cm/week with 0cm/week rainfall as the reference. Based on this exploratory analysis (df for lag of rainfall =5), lags 0-4 were selected for the final model.


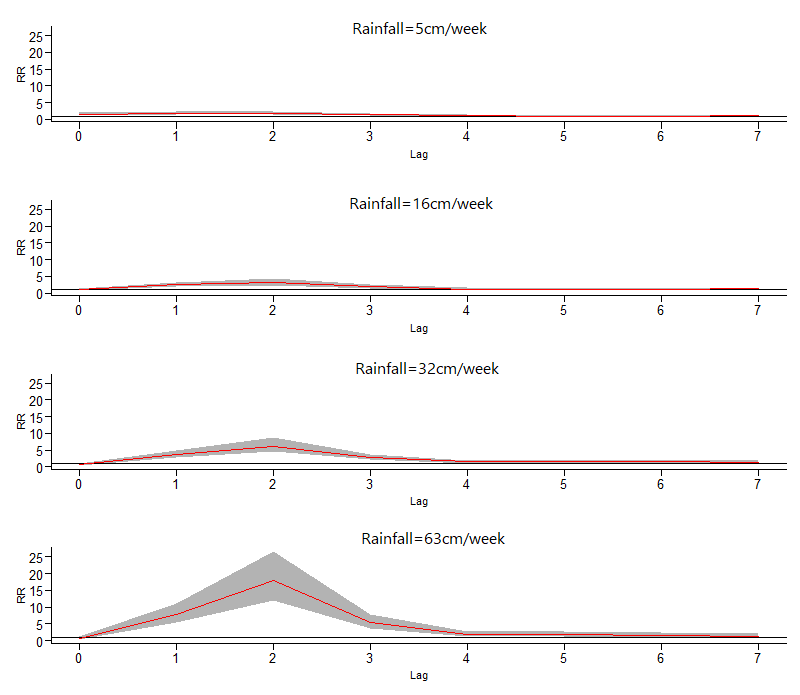

Supplement: S2 Fig — Each graph shows the relationship at lags 1 to 7 weeks for rainfall at 5, 16, 32 and 63 cm/week with 0cm/week rainfall as the reference. Based on this result, lags 0 to 4 weeks were selected for the final model. (DOCX) [file pntd.0006331.s009.docx]
