# Supplementary material for: The non-linear and lagged short-term relationship between rainfall and leptospirosis and the intermediate role of floods in the Philippines
Source: PLoS Negl Trop Dis. 2018 Apr 16;12(4):e0006331. doi: 10.1371/journal.pntd.0006331 (PMC5919665; doi:10.1371/journal.pntd.0006331)

**S3 Fig.** Relationships between rainfall and leptospirosis at the rainfall level of 32cm/week (right panel) and at lag of 2 weeks (left panel). (A) Subset 1, flood-unadjusted model (B) Subset 1, flood-adjusted model (C) Subset 2, flood-unadjusted model (D) Subset 2, flood-adjusted model. Refer to the caption of S1 Table for descriptions of these datasets.

(A)


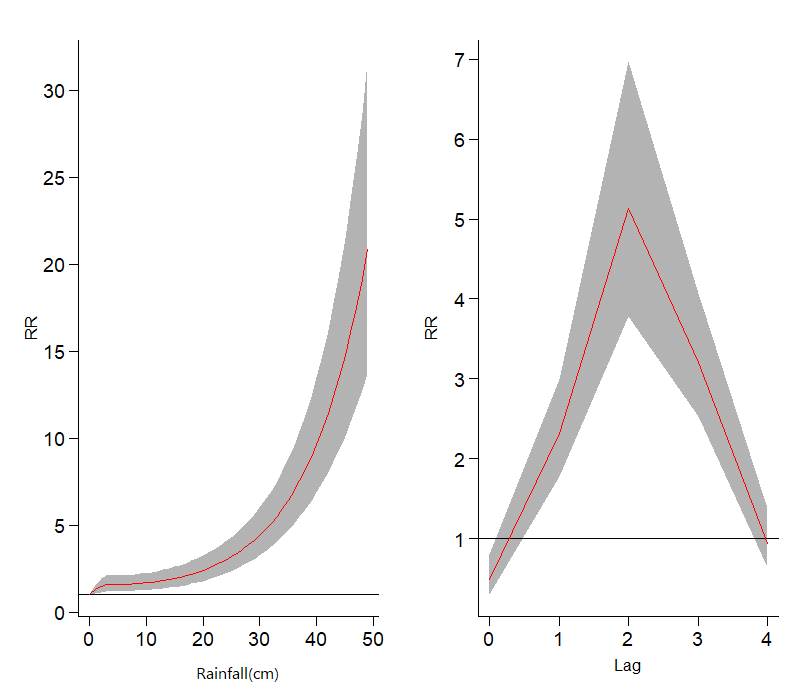


(B)


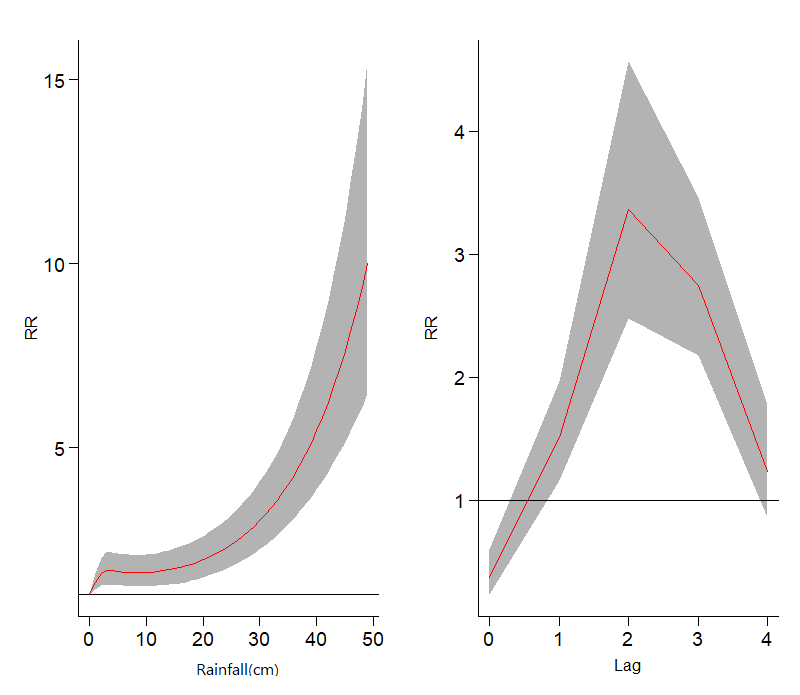


(C)


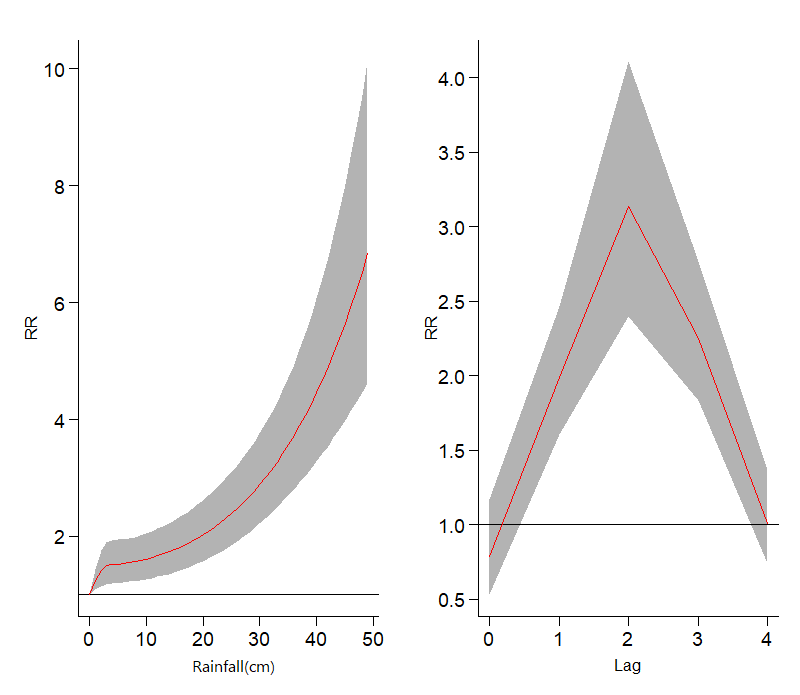


(D)


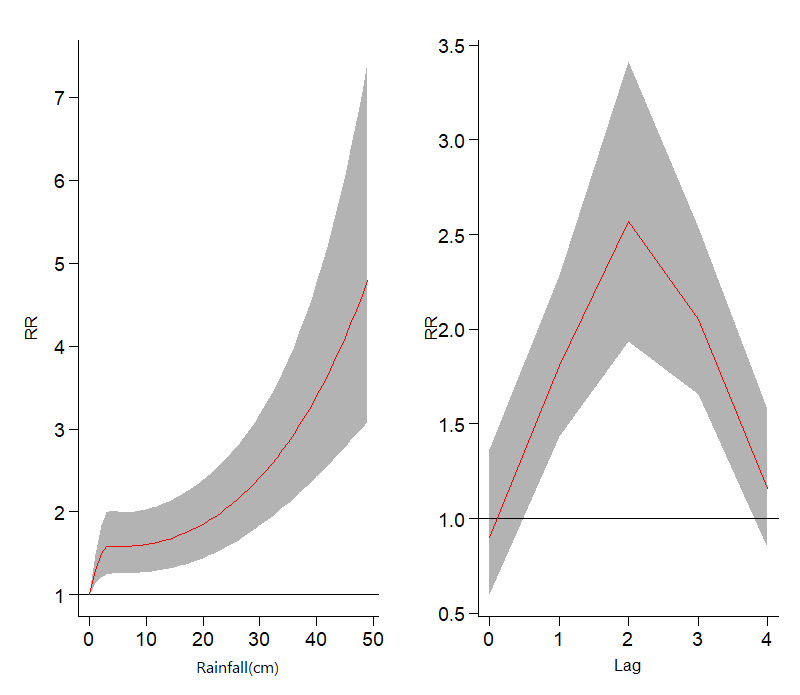

Supplement: S3 Fig — Relationships between rainfall and leptospirosis at the rainfall level of 32cm/week (right panel) and at lag of 2 weeks (left panel). (A) Subset 1, flood-unadjusted model (B) Subset 1, flood-adjusted model (C) Subset 2, flood-unadjusted model (D) Subset 2, flood-adjusted model. Refer to the caption of S1 Table for descriptions of these datasets. (DOCX) [file pntd.0006331.s010.docx]
